# Activation of Apoptotic Signal in Endothelial Cells through Intracellular Signaling Molecules Blockade in Tumor-Induced Angiogenesis

**DOI:** 10.1155/2015/908757

**Published:** 2015-08-04

**Authors:** Hossein Bazmara, M. Soltani, Kaamran Raahemifar, Mostafa Sefidgar, Majid Bazargan, Mojtaba Mousavi Naeenian, Ali Elkamel

**Affiliations:** ^1^Department of Mechanical Engineering, K.N.T. University of Technology, Tehran, Iran; ^2^Division of Nuclear Medicine, Department of Radiology and Radiological Science, School of Medicine, Johns Hopkins University, Baltimore, MD, USA; ^3^Electrical & Computer Department, Ryerson University, Toronto, ON, Canada; ^4^Department of Engineering and Technology, IKI University, Qazvin, Iran; ^5^Department of Chemical Engineering, University of Waterloo, Waterloo, ON, Canada

## Abstract

Tumor-induced angiogenesis is the bridge between avascular and vascular tumor growth phases. In tumor-induced angiogenesis, endothelial cells start to migrate and proliferate toward the tumor and build new capillaries toward the tumor. There are two stages for sprout extension during angiogenesis. The first stage is prior to anastomosis, when single sprouts extend. The second stage is after anastomosis when closed flow pathways or loops are formed and blood flows in the closed loops. Prior to anastomosis, biochemical and biomechanical signals from extracellular matrix regulate endothelial cell phenotype; however, after anastomosis, blood flow is the main regulator of endothelial cell phenotype. In this study, the critical signaling pathways of each stage are introduced. A Boolean network model is used to map environmental and flow induced signals to endothelial cell phenotype (proliferation, migration, apoptosis, and lumen formation). Using the Boolean network model, blockade of intracellular signaling molecules of endothelial cell is investigated prior to and after anastomosis and the cell fate is obtained in each case. Activation of apoptotic signal in endothelial cell can prevent the extension of new vessels and may inhibit angiogenesis. It is shown that blockade of a few signaling molecules in endothelial cell activates apoptotic signal that are proposed as antiangiogenic strategies.

## 1. Introduction

Cancer is the leading cause of death in North America and develops rapidly in other countries [1]. More than 85% of cancers involve solid tumors [2, 3]. At the beginning, tumors are avascular. In this stage, the tumor relies on diffusion for obtaining oxygen and nutrients. When a tumor grows further, diffusion mechanism is not able to supply required oxygen and nutrients to the tumor; therefore, a hypoxic region is formed inside the tumor. The tumor starts to release tumor angiogenic factors (TAFs) to induce nearby vessels to build new vessels toward the tumor and provide a dedicated source of oxygen and nutrients for it. After this step, the fatal vascular tumor growth phase starts. Formation of new capillaries from preexisting vessels is called angiogenesis. Multiple cells and factors are involved in angiogenesis; however, endothelial cells (ECs) play the main role in this process.

Blood vessels are constructed from ECs which are phenotypically inactive. When, due to some external stimuli like TAFs, EC changes to an active cell, it decides to stay inert, go, and/or grow. EC migration, proliferation, survival, and lumen formation are regulated by signals from extracellular matrix (ECM). Growth factors are the main sources of biochemical signals [4, 5], while biomechanical signals originate from interaction of EC with ECM or other ECs [6, 7]. After initial extension of sprouts, they may fuse together (anastomosis) and form closed flow pathways or loops. Blood flows in the closed loops and induces shear stress on the capillary walls. After anastomosis, shear stress induced by blood flow is the main source of biomechanical signals [8]. In the next stages, the loops elongate, fuse with other sprouts or loops, and create a network of capillaries, which circulates blood in the area that is closer to or inside the tumor. The capillary network has a vital role in further tumor growth [9–11].

**Figure 1:**
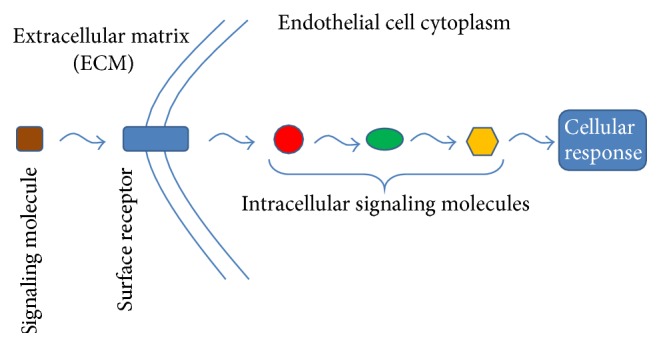
Signal transduction in EC. The surface receptors transduce the signals to the downstream molecules.

The environmental and flow induced signals regulate EC phenotype through activation of signaling cascades inside ECs. Several receptors and dozens of intracellular proteins are involved in signal transduction in the cascades. Each receptor is specialized to receive specific signals. Downstream of the receptor, there are intracellular proteins that transduce signals.  Figure 1 shows a schematic of signal transduction in an EC.

Each EC can be considered as a biological system with inputs and outputs. The system inputs are biochemical and biomechanical signals, while the outputs are EC phenotype and lumen formation. Inside an EC, interaction of intracellular signaling molecules regulates the relation between the inputs and outputs.

**Figure 2:**
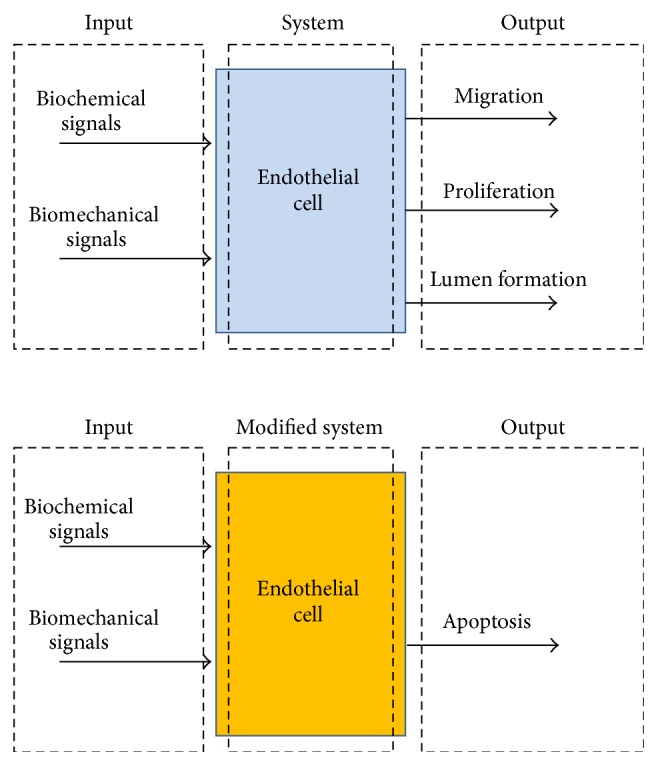
The relation between inputs and outputs in ECs. Due to modifications in the system (blockade of intracellular signaling molecules), its outputs are changed, which alters the cell fate and can inhibit angiogenesis.

EC migration, proliferation, and lumen formation are essential for successful angiogenesis. Intracellular signaling molecules play a vital role in regulation of the system output, so blocking one or a few signaling molecules changes the EC fate. If blockade of intracellular signaling molecules drives the cell to go for apoptosis, the process of angiogenesis stops. It is also possible to inhibit angiogenesis through inhibition of proliferation, migration, and lumen formation of ECs. Any interruption in or inhibition of angiogenesis reduces tumor growth or may even stop it. This strategy in cancer treatment is called antiangiogenic therapy.  Figure 2 shows a schematic of the relation between the system input and output for an EC with and without modifications.

Cellular behavior is controlled by signals that EC receives in the cell surface. Integrins, vascular endothelial (VE) Cadherin, and transmembrane receptors (such as tyrosine kinase receptors, G-protein-coupled receptors, tyrosine-kinase-associated receptors, and serine-threonine kinase receptors) are responsible for receiving EC environmental signals [17]; however, key events in sprouting angiogenesis are regulated by VEGF specific receptor tyrosine kinases (RTKs), integrins, and VE Cadherins [6]. Lumen formation is mainly controlled by the signals from integrin.

Mapping of environmental cues (inputs) to specific cell phenotypes (outputs) needs a model that takes into account intracellular molecules interactions and receptor cross talk. Regarding the intracellular signaling molecules behavior, Boolean network can be used to model signal transduction networks. Application of Boolean network in biological and medical modeling dates back to the 1960s when Stuart Kauffman used Boolean network to model genetic regulatory networks [27]. Other theoretical studies in biology and medicine have used Boolean networks in morphogenesis [28], yeast cell cycle dynamics [29, 30], signal transduction networks [6, 7, 31], major depressive disorder [32], and study on permeabilization of mitochondrial outer membrane [33].

Li et al. used a Boolean model to develop a dynamic model of guard cell abscisic acid signaling [31]. In the context of angiogenesis, Bauer et al. constructed a Boolean network model of critical signaling pathways in ECs [6, 7]. Their model establishes a map between an EC phenotype and signals that an EC receives from the environment. The map between the EC input signals and phenotype can play a vital role in multiscale models of sprouting angiogenesis [34, 35].

In this study, two different stages are considered for vascular development during sprouting angiogenesis. Prior to anastomosis, ECs receive the biochemical and biomechanical signals from ECM. The relation between environmental signals and EC lumen formation has not been considered in the previous models. To build a model for EC phenotype and lumen formation prediction, critical signaling pathways of EC migration, apoptosis, proliferation, and lumen formation are combined. After anastomosis, blood flow induced shear stress is the main regulator of EC phenotype. Different signaling cascades are activated in each stage (before and after anastomosis). Driving ECs to go for apoptosis is possible through blockade of intracellular signaling molecules. Effect of intracellular signaling molecules blockade is investigated for each signaling cascade (before and after anastomosis). Activation of apoptotic signal in ECs inhibits further vascular development and may help to stop tumor growth.

## 2. Materials and Methods

The basic step in development of a model for EC phenotype determination is to obtain the signaling cascades and logical rules of interaction (Boolean dependence relations) between intracellular signaling molecules. In the next step, the signaling cascades and the Boolean dependence relations are used in a Boolean network model to construct the network.

### 2.1. Signaling Cascade of EC before Anastomosis (Without Blood Flow)

During single sprout development, biochemical and biomechanical signals from ECM regulate EC phenotype. Bauer et al. proposed a signaling cascade for EC phenotype determination in single sprouts [6]. In addition to growth and migration, ECs should acquire luminal compartment during vessel development. In this section, the signaling pathway of lumen formation is combined with the signaling pathway proposed by Bauer et al. for phenotype determination [6] to obtain a complete signaling cascade before anastomosis.

Lumen formation is a key step in vascular morphogenic events. An increasing number of studies try to explain acquirement of lumenal compartments in blood vessels [36–43]. Using* in vivo* and* in vitro* models, signaling cascade of lumen formation has been investigated [36, 38, 40–42, 44–47]. Interaction of integrins and extracellular matrix activates a cascade of events inside ECs. Downstream of integrin signaling, activation of Cdc42, Rac1, and Src plays a substantial role in vascular lumen formation [38, 45, 48, 49]. Cdc42, Par6, Par3, membrane type 1-matrix metalloproteinase (MT1-MMP), and integrin coassociate to control EC lumen formation [50].

Downstream of Cdc42 and Rac1, other proteins are activated to transduce signals and modulate cell cytoskeleton. Small GTPase Rac1 activates Pak2. Cdc42 activates Pak2 and Pak4. Pak2 and Pak4 are also activated by protein kinase C (PKC), especially isoform PKC*ε* [42, 51]. Cdc42 activates Par3, Par6, and PKC*ζ*. Activation of Src, Pak2, Pak4, Par3, Par6, and PKC*ζ* is required for lumen formation.

**Figure 3:**
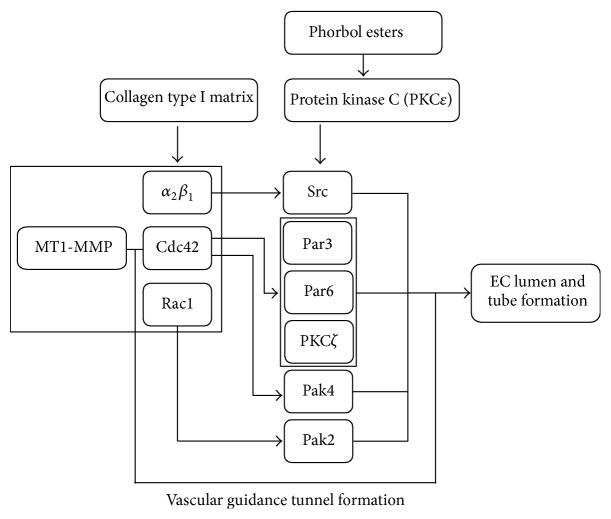
Schematic of general signaling network for EC lumen and tube formation. Multiple intracellular proteins are involved in the network. Lumen formation is mainly coordinated by integrin, Rac1, Cdc42, Src, Par3, Par6, PKC*ζ*, Pak2, and Pak4. Arrows indicate activation signal.

Based on the available* in vivo* and* in vitro* experiments, Davis et al. proposed a signal transduction pathway for EC lumen and tube formation [42]. A schematic of the proposed signaling pathways is shown in Figure 3.

In the proposed signaling pathway for lumen formation in Figure 3, phorbol esters (which are known to enhance angiogenesis and lumen formation) are used as an external activator of PKC*ε*. It is assumed that, in the process of sprouting angiogenesis, external signals exist to activate PKC*ε*. MMP establishes vascular guidance tunnel that is essential for tube formation, so the role of MMP is neglected in signaling cascade of lumen formation.

**Figure 4:**
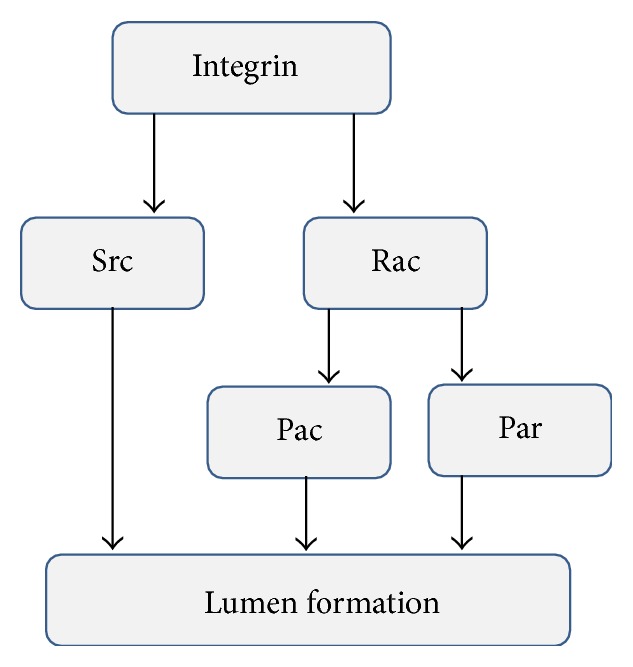
Simplified signaling network for lumen formation. The network in Figure 1 is simplified for use in the main signaling network. Cdc42 and Rac1 are integrated into a single node (Rac). The Rac activation mechanism by integrin is not expanded in this figure. Par3, Par6, and PKC*ζ* are integrated into Par. Pak2 and Pak4 are integrated into Pak. Since the activation of Pak and Par nodes is parallel, they are also integrated in the main signaling network.

To incorporate lumen formation signaling pathway into the phenotype determination signaling pathway, the signaling pathway in Figure 3 is simplified through nodes information integration. Rac1 and Cdc42 are integrated in a unified node without loss of information [6]. Pak2 and Pak4 are also integrated into a single node (Pak) and Par3 and Par6 into Par. Pak and Par proteins are also integrated into a single node. These assumptions, however, do not cause any loss of information.  Figure 4 shows a simplified signaling pathway of lumen formation.

**Figure 5:**
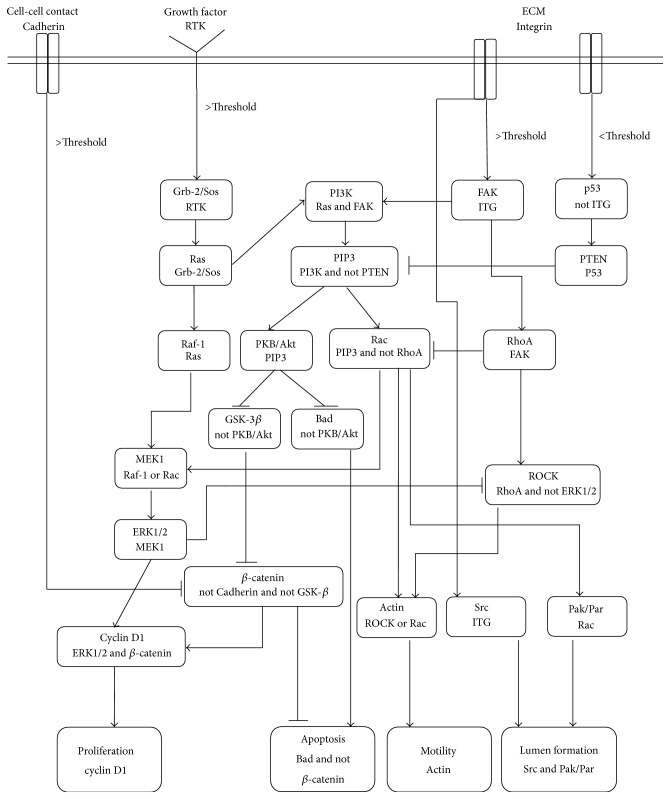
Signaling cascade of EC phenotype determination and lumen formation. The double line in this figure represents cell boundary schematically. The environmental signals are transmitted into the cell through cell surface receptors. Each box represents an intracellular signaling molecule (or integration of a few molecules). The first line in each box is its title, and the second line is its Boolean dependence relation or activation rule. An arrow indicates an activation signal while a hammerhead indicates an inhibition. The intersections of horizontal and vertical lines do not represent any type of connection. The four lower boxes indicate cell phenotype and lumen formation. The activation of each phenotype and lumen formation depends on the combination of the inputs (cell surface receptor status).

The incorporation of signaling pathway of lumen formation into the signaling pathways of cell phenotype determination is performed and the result is presented in Figure 5.

In the schematic presented in Figure 5, receptors cross talk and Boolean dependence relations between intracellular molecules are shown. In this schematic, an arrow indicates an activation signal while a hammerhead indicates inhibition signal. In each box or node, the first line is the node title (signaling molecule) and the second line is its Boolean dependence relation with other nodes. The Boolean dependence relations determine activation or deactivation of nodes. For example, Grb-2/Sos activates Ras. Ras activates Raf-1 and contributes to the activation of PI3K (with FAK).

Three main inputs from the surface receptors of ECs are considered for this network. In the first input, cell-cell contact or Cadherin is representative of VE Cadherin in endothelial cells. The second input is RTK and represents chemical signals from VEGF in the domain. The third one, integrin, is responsible for sensing the amount of attachment of ECs to ECM molecules such as matrix fibers. Activation or deactivation of any receptor and the downstream effectors directly affects cell response. In ECs, the response is cell phenotype, that is, proliferation, apoptosis, and/or motility or lumen formation.

Rac and Rho are main agents in cross talk between signaling pathways. Different feedback mechanisms for interplay between Rac and Rho are reported in the literature [52–55]; however, no definitive mechanism for interaction of Rac with other signaling molecules especially Rho is mentioned in the literature. Bauer and Rohlf [7] studied the effect of different feedback mechanisms between Rac and Rho. In the model developed here, inhibitory effect of Rho on Rac is utilized.

Figure 5 shows the signaling cascade of ECs before blood flow. The Boolean network model is used to map environmental signals (inputs) to cell phenotype and lumen formation (outputs). The reader is referred to Bauer and Rohlf for the descriptions and details of the Boolean network model [7].

### 2.2. Signaling Cascade of ECs after Anastomosis (With Blood Flow)

EC function and phenotype are affected by shear stress induced by blood flow. Cultured ECs reorient their longitudinal axis according to the streamlines of the flow. The reorientation will reduce the effective shear stress on ECs [21]. Several studies show that shear stress has a pivotal role in EC survival and prevention of apoptosis [56, 57]. There is also evidence that shear stress impacts EC proliferation [12, 58]. In wound healing, laminar shear stress enhances EC migration [59, 60].

At the intracellular scale, experimental studies determine the role of cell surface receptor and intracellular signaling molecules in signaling cascade of shear stress. Integrin is involved in shear stress mechanotransduction [61] and activation of receptor tyrosine kinases (RTKs) [62]. Activation of integrin activates FAK, paxillin, c-Src, Fyn, and P130, which leads to activation of Ras-ERK pathway [13, 14]. The ERK pathway is involved in cell growth and proliferation [63]. Shear stress also activates RTKs including VEGFR2 and Tie2. The activation of RTKs is independent of VEGF [19, 20, 64, 65]. Activation of RTKs activates MAPK pathways including ERK, JNK, PI3K, and Akt through activation of Ras. These pathways are the main regulator for cell survival and inhibition of apoptosis [8, 12]. Moreover, shear stress causes rapid tyrosine phosphorylation of PECAM-1 [16]. Activation of ERK is dependent on PECAM-1 [66]. PECAM-1, VEGFR2, and VE Cadherin form a complex mechanosensory system. This system has a critical role in transduction of shear stress signals [67]. PECAM-1 and VE Cadherin are necessary for shear stress activation of integrin [15].

**Figure 6:**
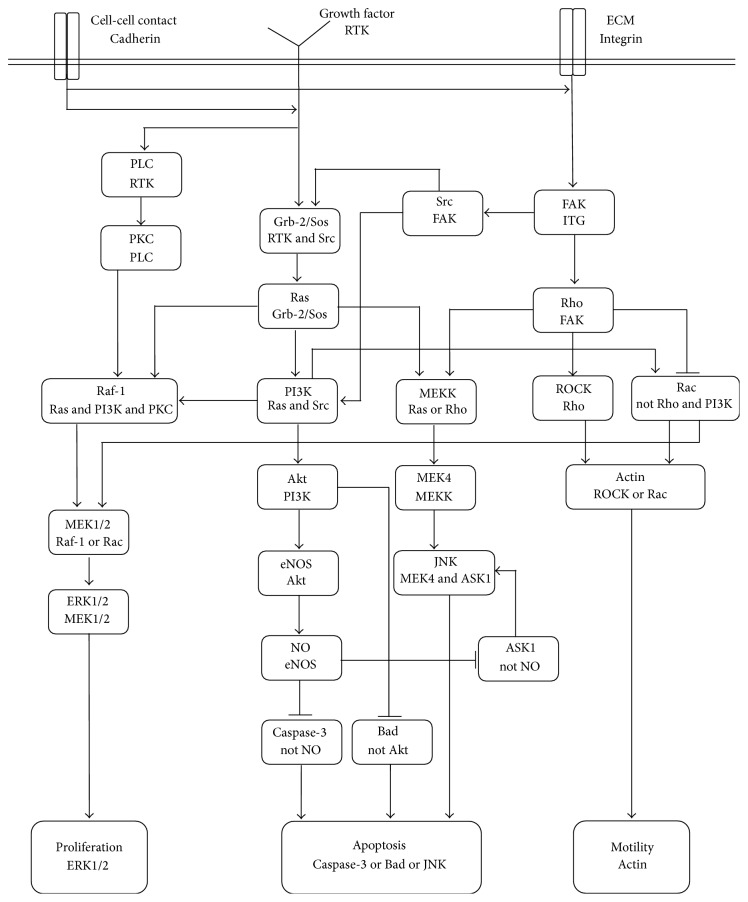
Signaling cascade of shear stress activation of EC. The double line in the figure represents cell boundary schematically. The shear stress induced signals are transmitted into the cell through cell surface receptors. Each box represents an intracellular signaling molecule. The first line in each box is the box title. The second line is the Boolean dependence relation or activation rule of the box. An arrow indicates an activation signal while a hammerhead indicates an inhibition signal. The intersections of horizontal and vertical lines do not represent any type of connection. The three lower boxes indicate cell phenotype.

Different candidates for shear stress sensors in ECs were introduced; however, as mentioned before, main events in ECs are regulated by RTK, integrin, and VE Cadherin. Based on the available experimental data, a signaling cascade activated by shear stress is proposed here and shown in Figure 6.

Table 1 outlines the Boolean dependence relation of the network shown in Figure 6 and the corresponding references for each relation.

## 3. Results and Discussion

The signaling cascades in Figures 5 and 6 show the intracellular molecules interactions during vascular development before and after presence of blood flow in capillaries. A Boolean network model is used to analyze the signaling cascades. The EC system inputs are receptors status and the outputs are cell phenotype. To investigate the effect of intracellular signaling molecules blockade on system output, the intracellular signaling molecules statuses are set “off” one by one and the system output is recorded. When the status of an intracellular signaling molecule is set “off,” it means that it cannot transduce the signals to the downstream molecules.

In each case, there are three system inputs, that is, RTK, integrin, and VE Cadherin. Our analyses show that, with and without blood flow, when RTK or integrin signals are not active, the apoptosis is the system output. This is the first general result in activation of apoptotic signal, which requires the blockade of the surface receptors of EC. To investigate the effect of intracellular signaling molecules blockade in cell fate, it is assumed that both integrin and RTK signals are active in the rest of the analysis.

### 3.1. Intracellular Signaling Molecules Blockade before Anastomosis (Without Blood Flow)

Before anastomosis and blood flow start, the main regulator of EC phenotype is biochemical and biomechanical signals from ECM. Targeted inhibition of specific signaling molecules can alter the cell phenotype, thus inhibiting angiogenesis. In this method, using blocking antibodies or technologies to suppress the expression of individual genes, specific signaling molecules are inhibited. The change may make the ECs unable to form lumen, proliferate, and migrate. Ideally, the ECs undergo apoptosis.

**Figure 7:**
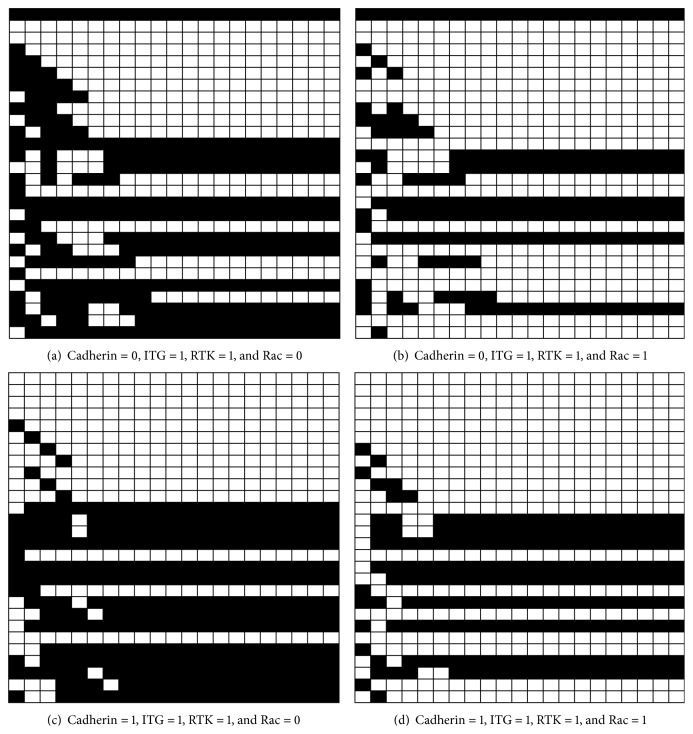
Boolean network model output when no molecule is inhibited. The combinations of the input signals are shown below each subfigure. The iterations run from left to right in each subfigure. The white box corresponds to 1 (active) and the black box corresponds to 0 (inactive). In each subfigure, the 1st row is Cadherin signal status, the 2nd row is RTK signal status, and the 3rd row is integrin (ITG) signal status. The Rac status is shown in the 12th row. The input signals are constant during iterations. The initial states of other nodes in the network are chosen randomly. Nodes states change during the solution until a converged set of states is obtained for nodes. The last four rows are outputs that are proliferation, apoptosis, motility, and lumen formation, respectively.

A Boolean network model is used to analyze the signaling cascade in Figure 5. As a sample, the model output for the case with no blocked molecules is shown in Figure 7.

Each surface receptor and box in Figure 5 is represented by a node in the leftmost column in Figure 7. Nodes 1 to 3 in the column correspond to the three surface receptors, that is, cell-cell contact, RTK, and integrin, respectively. Nodes 4 to 24 are intracellular signaling molecules. The last four nodes in the column determine cell phenotype and lumen formation. Nodes 25 to 28 correspond to cell responses that are proliferation, no apoptosis, migration, and lumen formation.

The first column in Figure 7 is the initial condition of the nodes. The initial conditions for nodes 1 to 3 are the input signals and for nodes 4 to 28 are set randomly. Iterations run from left to right and the rows in Figure 7 show nodes evolutions during iterations. After 20 iterations, the last column shows the output signals. In the first eight iterations, columns 1 to 8, the nodes change their states until a converged state is obtained in iteration 9 and stays until the end of the solution.

It should be mentioned that the network output (state of nodes 25 to 28 in the last iteration) is independent of initial conditions of intracellular signaling molecules (nodes 4 to 28). This is verified by multiple runs from several initial condition sets, which all converge to a unique set of phenotypes.

The state of Rac plays a central role in receptor cross talk in this model. This is verified by activating or deactivating this node, which results in different cell phenotypes [6]. Considering two possible states for Rac (activation and deactivation) and three environmental input signals, there are sixteen possible states for the model initial conditions.

EC phenotypes alteration due to inhibition of intracellular signaling molecules is presented in Table 2.

The four combinations of Cadherin and Rac are shown in the top row of Table 2. The last column is EC fate. Wild type is obtained when no molecule is blocked. When the apoptotic signal is activated, the cell fate is apoptosis, regardless of other activated signals. As can be concluded from Table 2, blockade of Grb2/Sos, Ras, PI3K, PIP3, PKB/Akt, and FAK drives the ECs to go for apoptosis before blood flow.

Blockade of a few molecules like Raf-1 and MEK1 does not lead to a specific result; that is, the cell fate changes, but it cannot help to inhibit angiogenesis. In some cases, blockade of signaling molecules changes the cell fate and makes them unable to go and/or grow. Making the cell unable to migrate, proliferate, or form lumen may help to inhibit angiogenesis.

### 3.2. Intracellular Signaling Molecules Blockade after Anastomosis (With Blood Flow)

After anastomosis, blood flow starts in the capillaries and the shear stress induced by blood flow is the main regulator of EC phenotype. Similar to the case without blood flow, both integrin and RTK are assumed to be active during analysis; else, the apoptotic signal is produced. The signaling cascade of Figure 6 is analyzed with Boolean network model and the results of intracellular signaling molecules blockade are presented in Table 3. In this case, Rac is not considered as an independent signal.

The structure of Table 3 is similar to Table 2. Though the blockade of signaling molecules changes the cell fate, only blockade of a few molecules produces apoptotic signal. Blockade of Grb2/Sos, FAK, Ras, PI3K, Akt, eNOS, and NO drives the ECs to go for apoptosis after blood flow. Making the cells unable to proliferate and migrate is also possible after blood flow.

## 4. Conclusion

Considering each EC as a system with inputs and outputs, specific strategies are tested to inhibit angiogenesis. A Boolean network model of receptor cross talk for cell phenotype determination including lumen formation is used. This is, to our knowledge, the first study that presents a comprehensive model of cell phenotype determination and lumen formation based on environmental cues. Effect of inhibition of each intracellular signaling molecule and possible antiangiogenic effects are investigated in this study. The model predicts that inhibition of a few intracellular signaling molecules can be used to inhibit angiogenesis, thus posing a strategy to achieve the antiangiogenic effects.

## Figures and Tables

**Table 1:** Nodes, node dependence relations, and corresponding references. The input state combination in each node determines the update rule for that node.

Node	Dependence relation	Reference
Integrin	VE Cadherin and flow	[12–15]
RTK	Flow	[16]
PLC	RTK	[8, 17]
Grb-2/Sos	RTK and Src	[18]
FAK	Integrin	[15, 17, 19, 20]
Src	FAK	[17]
PKC	PLC	[17]
Ras	Grb-2/Sos	[8, 18, 19]
Rho	FAK	[17]
PI3K	Ras and Src	[6, 7]
MEKK	Ras or Rho	[8]
ROCK	Rho	[17]
Rac	Not Rho and PI3K	[6, 7]
Raf-1	Ras and PI3K and PKC	[8, 17, 21, 22]
MEK1/2	Raf-1 or Rac	[17, 23]
Akt	PI3K	[16, 17]
MEK4	MEKK	[8]
Actin	ROCK or Rac	[17]
ERK1/2	MEK1/2	[8, 17]
eNOS	Akt	[16, 17]
JNK	MEK4 and ASK1	[8, 24]
NO	eNOS	[16, 17]
ASK1	Not NO	[25]
Caspase-3	Not NO	[24]
Bad	Not Akt	[24]
Proliferation	ERK1/2	[17, 26]
Apoptosis	Caspase-3 or Bad or JNK	[24]
Migration	Actin	[17]

**Table 2:** EC phenotype classification and fate for inhibition of each intracellular signaling molecule before blood flow. The four combinations of Cadherin and Rac are shown in the top row. The row corresponding to “None” shows the cell phenotype when no molecule is blocked and EC fate is considered as wild type. The third row shows abbreviation of each phenotype. P stands for proliferation, A stands for apoptosis, M stands for motility, and L stands for lumen formation. One and 0 in each row determine activation and deactivation of each cell phenotype. The last column is EC fate.

Blocked signaling molecule	ITG = 1, RTK = 1, Cadherin = 0, Rac = 0				ITG = 1, RTK = 1, Cadherin = 0, Rac = 1				ITG = 1, RTK = 1, Cadherin = 1, Rac = 0				ITG = 1, RTK = 1, Cadherin = 1, Rac = 1				EC fate
	EC phenotype classification																
	P	A	M	L	P	A	M	L	P	A	M	L	P	A	M	L	
None	**1**	**0**	**0**	**0**	**1**	**0**	**1**	**1**	**0**	**0**	**0**	**0**	**0**	**0**	**1**	**1**	Wild type
Grb-2/Sos	0	1	0	0	0	1	0	0	0	1	0	0	0	1	0	0	Apoptosis
Ras	0	1	0	0	0	1	0	0	0	1	0	0	0	1	0	0	Apoptosis
Raf-1	0	0	1	0	1	0	1	1	0	0	1	0	0	0	1	1	Not specified
MEK1	0	0	1	0	0	0	1	1	0	0	1	0	0	0	1	1	Not specified
ERK1/2	0	0	1	0	0	0	1	1	0	0	1	0	0	0	1	1	Not specified
PI3K	0	1	0	0	0	1	0	0	0	1	0	0	0	1	0	0	Apoptosis
PIP3	0	1	0	0	0	1	0	0	0	1	0	0	0	1	0	0	Apoptosis
PKB/Akt	0	1	0	0	0	1	0	0	0	1	0	0	0	1	0	0	Apoptosis
Rac1	1	0	0	0	1	0	0	0	0	0	0	0	0	0	0	0	Unable to migrate
GSK-3*β*	1	0	0	0	1	0	1	1	0	0	0	0	0	0	1	1	Not specified
Bad	1	0	0	0	1	0	1	1	0	0	0	0	0	0	1	1	Not specified
*β*-catenin	0	0	0	0	0	0	1	1	0	0	0	0	0	0	1	1	Unable to proliferate
FAK	0	1	0	0	0	1	0	0	0	1	0	0	0	1	0	0	Apoptosis
p53	1	0	0	0	1	0	1	1	0	0	0	0	0	0	1	1	Wild type
PTEN	1	0	0	0	1	0	1	1	0	0	0	0	0	0	1	1	Wild type
RhoA	1	0	0	0	1	0	1	1	0	0	0	0	0	0	1	1	Wild type
ROCK	1	0	0	0	1	0	1	1	0	0	0	0	0	0	1	1	Wild type
Actin	1	0	0	0	1	0	0	1	0	0	0	0	0	0	0	1	Unable to migrate
Cyclin D1	0	0	0	0	0	0	1	1	0	0	0	0	0	0	1	1	Unable to proliferate
Src	1	0	0	0	1	0	1	0	0	0	0	0	0	0	1	0	Unable to form lumen
Pak/Par	1	0	0	0	1	0	1	0	0	0	0	0	0	0	1	0	Unable to form lumen

**Table 3:** EC phenotype classification and fate for inhibition of each intracellular signaling molecule after blood flow. The row corresponding to “None” shows the cell phenotype when no molecules are blocked and EC fate is considered as wild type. The third row shows the abbreviation of each phenotype. P stands for proliferation, A stands for apoptosis, and M stands for motility. One and 0 in each row determine activation and deactivation of each cell phenotype. The last column is EC fate.

Blocked signaling molecule	ITG = 1, RTK = 1, Cadherin = 0			ITG = 1, RTK = 1, Cadherin = 1			EC fate
	EC phenotype classification						
	P	A	M	P	A	M	
None	**0**	**1**	**0**	**1**	**0**	**1**	Wild type
PLC	0	1	0	0	0	1	Unable to proliferate
PKC	0	1	0	0	0	1	Unable to proliferate
Grb-2/Sos	0	1	0	0	1	1	Apoptosis
Src	0	1	0	0	1	1	Apoptosis
FAK	0	1	0	0	1	0	Apoptosis
Ras	0	1	0	0	1	1	Apoptosis
Rho	0	1	0	1	0	1	Wild type
Raf-1	0	1	0	0	0	1	Unable to proliferate
PI3K	0	1	0	0	1	1	Apoptosis
MEKK	0	1	0	1	0	1	Wild type
ROCK	0	1	0	1	0	0	Unable to migrate
Rac	0	1	0	1	0	1	Wild type
MEK1/2	0	1	0	0	0	1	Unable to proliferate
Akt	0	1	0	1	1	1	Apoptosis
MEK4	0	1	0	1	0	1	Wild type
Actin	0	1	0	1	0	0	Unable to migrate
eNOS	0	1	0	1	1	1	Apoptosis
JNK	0	1	0	1	0	1	Wild type
ERK1/2	0	1	0	0	0	1	Unable to proliferate
NO	0	1	0	1	1	1	Apoptosis
ASK1	0	1	0	1	0	1	Wild type
Caspase-3	0	1	0	1	0	1	Wild type
Bad	0	1	0	1	0	1	Wild type
